# Disparities in Breast Cancer Characteristics Among Syrian Migrants and Jordanian Women in the Jordan Cancer Registry from 2010 to 2016

**DOI:** 10.1001/jamanetworkopen.2023.25197

**Published:** 2023-07-24

**Authors:** Aditi Hazra, Andreas Ullrich, Omar Nimri

**Affiliations:** 1 Division of Preventive Medicine, Department of Medicine, Brigham and Women’s Hospital and Harvard Medical School, Boston, Massachusetts; 2 Department Gynaecology Charité University, Charité Universitätsmedizin, Berlin, Germany; 3 Disease Prevention and Control Directorate, Jordan Center for Disease Control, Amman, Jordan

## Abstract

This case series analyzes breast cancer characteristics of women diagnosed with breast ancer in the Jordan Cancer Registry by Syrian migrant status to determine inequities.

## Introduction

Although early detection of breast cancer has improved, there are disparities related to the effects of war.^1,2,3,4^ Refugees surviving the climate and conflict polycrisis or gender-based violence (GBV) as a weapon of war have an increased risk of cancer mortality compared with the host population.^2,5^ We analyzed breast cancer characteristics in the Jordan Cancer Registry (JCR) by Syrian migrant status (age-standardized breast cancer incidence, 95.1 per 100 000^1,4^ individuals) to elucidate inequities.

## Methods

This case series followed the Strengthening the Reporting of Observational Studies in Epidemiology–Equity (STROBE–Equity) reporting guideline. This study was approved by the Jordan Cancer Registry (JCR) and exempt from informed consent because identifiable information was not included.

We conducted a retrospective observational case series of 7891 women diagnosed with breast cancer (*International Statistical Classification of Diseases and Related Health Problems, Tenth Revision*, C50) from January 1, 2010, to December 31, 2016, in the JCR. Data were collected from all hospitals and pathology and hematology laboratories through case findings, visits to medical facilities, and the return of case forms to JCR. Migrants needed identification cards from the United Nations High Commission for Refugees (UNHCR) or the Ministry of the Interior to receive health care. Women were classified as Syrian migrants or Jordanians using nationality codes.

We evaluated breast cancer characteristics, including age, date of diagnosis, smoking, marital status, tumor characteristics (ie, grade, stage), and clinical covariates. Additional information about our Methods can be found in the eMethods in Supplement 1.

R Studio version R 4.2.2 (R Project for Statistical Computing) was used for descriptive analyses. A 2-sided *P* < .05 was considered significant. Data were analyzed from November 2022 to March 2023.

## Results

This study included a total of 7891 women (375 [4.8%] Syrian women who were migrants and 7516 [95.2%]) Jordanian women. The Table shows the characteristics of the study population. The median (IQR) age at breast cancer presentation was 49 (40-60) years among Syrian women who were migrants and 51 (43-61) years among Jordanian women (Kruskal-Wallis *P* <.001). Most women were married (315 Syrians [84.0%] and 6056 Jordanians [80.6%]; Fisher exact test *P* <.001) and never smokers (221 Syrians [58.9%] and 5067 Jordanians [67.4%]; *P* = .11).

**Table.** Clinical Characteristics of Breast Cancer, Diagnosed From 2010-2016, Among Syrian Migrants and Jordanian Women in Jordan

Characteristics	Women, No. (%)		*P* value
	Syrian	Jordanian	
Total	375 (4.8)	7516 (95.2)	NA
Age at diagnosis, y			
Median (IQR)	49 (40-60)	51 (43-61)	<.001
≤40	94 (25.07)	1378 (18.33)	.005
≤49	100 (26.67)	2101 (27.95)	
≥50	181 (48.27)	4037 (53.71)	
Year of diagnosis			
2010	14 (3.73)	973 (12.95)	<.001
2011	14 (3.73)	934 (12.43)	
2012	26 (6.93)	998 (13.28)	
2013	117 (31.20)	1043 (13.88)	
2014	69 (18.40)	1172 (15.59)	
2015	69 (18.40)	1133 (15.07)	
2016	66 (17.60)	1263 (16.80)	
Marital status			
Single	16 (4.27)	460 (6.12)	<.001
Married	315 (84.00)	6056 (80.57)	
Widowed	0	9 (0.12)	
Divorced	3 (0.80)	169 (2.25)	
Other	9 (2.40)	562 (7.48)	
Unknown^a^	32 (8.53)	257 (3.42)	
Smoking status			
Never smoker	221 (58.93)	5067 (67.42)	.11
Current smoker	15 (4.00)	598 (7.96)	
Past smoker	5 (1.33)	137 (1.82)	
Unknown^a^	131 (34.93)	1686 (22.43)	
SEER summary stage at diagnosis			
In situ	4 (1.07)	296 (3.94)	<.001
Localized	29 (7.73)	1204 (16.02)	
Regional direct extension	31 (8.27)	635 (8.45)	
Regional lymph node	41 (10.93)	1529 (20.34)	
Regional direct extension and lymph node	75 (20.00)	1111 (14.78)	
Regional NOS	3 (0.80)	125 (1.66)	
Distant metastasis	88 (23.47)	884 (11.76)	
Unknown in registry^a^	104 (27.73)	1719 (22.87)	
Not recorded^a^	0	13 (0.17)	
Laterality	375	7516	
Right	160 (42.67)	3392 (45.13)	.33
Left	168 (44.80)	3708 (49.33)	
One side, unknown	0	18 (0.24)	
Bilateral^b^	9 (2.40)	108 (1.44)	
Unknown^a^	38 (10.13)	290 (3.86)	
Tumor grade			
I	5 (1.33)	350 (4.66)	.34^c^
II	93 (24.80)	2893 (38.49)	
III	76 (20.27)	2215 (29.47)	
IV	0	21 (0.28)	
Null cell	0	11 (0.15)	
Unspecified	201 (53.60)	2025 (26.94)	
Tumor behavior			
In situ	3 (0.80)	271 (3.61)	<.001
Invasive	372 (99.20)	7245 (96.39)	
Morphology (code)			
Neoplasm, malignant (8000)	13 (3.47)	106 (1.41)	<.001
Carcinoma, NOS (8010)	59 (15.73)	540 (7.18)	
Adenocarcinoma, NOS (8140)	10 (2.67)	46 (0.61)	
Infiltrating ductal carcinoma	263 (70.13)	5725 (76.17)	
Lobular carcinoma, NOS (8500)	16 (4.27)	525 (6.99)	
Infiltrating ductal and lobular carcinoma (8522)	0	102 (1.36)	
Other	14	574	

Abbreviations: NA, not applicable; NOS, not otherwise specified; SEER, surveillance, epidemiology, and end results.

^a^ *P* value presented for cases with complete data (unknown category excluded).

^b^ Syrian women younger than 49 years were more likely to have bilateral breast cancer (*P* value = .049).

^c^ Fisher exact test.

From 2010 to 2016, 300 women (4.0%) were diagnosed with in situ breast carcinoma; 1233 (16.4%) with localized disease, 666 (8.9%) with regional disease; 1570 (20.9%) with regional lymph node(s) involved; 1186 (15.8%) with regional by both direct extension and lymph node(s); and 972 (12.3%) with distant metastasis. Syrian women who were migrants were more likely than Jordanian women to be diagnosed with distant metastasis (88 [23.5%] for Syrian women vs 884 [11.8%] for Jordanian women; χ^2^ *P* value <.001; crude odds ratio, 2.66 [95% CI, 2.02-3.49]). Distribution of tumor grade was different for Syrian migrant women (grade I, 5 [1.3%]; grade II, 93 [24.8%]; grade III, 76 [20.3%]) and Jordanian women (grade I, 350 [4.7%]; grade II, 2893 [38.5%] grade III, 2215 [29.5%]) (Fisher exact test *P* value = .34). However, the tumor grade was missing for 201 Syrian women who were migrants (53.6%) but only 2025 Jordanian women (26.9%). Syrian women aged 49 years or younger were more likely to present with bilateral breast cancer (Fisher exact test *P* value = .049).

## Discussion

We found disparities in bilateral breast cancer at younger ages and late-stage^1^ breast cancer presentation in Syrian migrant women compared with Jordanian women. Our findings are important because refugees with late-stage cancer or poor prognoses were less likely to receive UNHCR support^3^ (missing treatment data). Strengths of the quantitative study include tumor characteristics on 7891 breast cancer cases among Syrian and Jordanian women from a national, population-based cancer registry. Limitations include the aggregation of forced migrants and other migrants under the term migrants, missing data on stage, grade, treatment, bias (camp-urban gradient), GBV/trauma,^5^ potential misclassification of stage-at-diagnosis,^6^ underreporting of cases,^4^ and generalizability.

Multistakeholder support to scale early detection (approximately 4% in situ carcinoma among Jordanians), integrate cancer control in crises in national plans, digitize and complete case ascertainment in registries,^6^ and access to multimodality treatment^1,2,3^ may improve survival. Global shared responsibility is needed for the advancement of equity across the cancer continuum and stages, local development with nationals and refugees,^6^ gender equality, and peacebuilding efforts in protracted polycrisis settings (eg, due to drought, war, and earthquakes).

## Notes

Supplement 1.
eMethods.

Supplement 2.
Data Sharing Statement
