# Supplementary material for: Disparities in Breast Cancer Characteristics Among Syrian Migrants and Jordanian Women in the Jordan Cancer Registry from 2010 to 2016
Source: JAMA Netw Open. 2023 Jul 24;6(7):e2325197. doi: 10.1001/jamanetworkopen.2023.25197 (PMC10366694; doi:10.1001/jamanetworkopen.2023.25197)
Supplement: Supplement 1. — eMethods. [file jamanetwopen-e2325197-s001.pdf]

Supplemental Online Content

Hazra A, Ullrich A, Nimri O. Disparities in breast cancer characteristics among Syrian migrants and Jordanian women in the Jordan Cancer Registry from 2010 to 2016. *JAMA Netw Open*. 2023;6(7):e2325197. doi:10.1001/jamanetworkopen.2023.25197

eMethods.

This supplemental material has been provided by the authors to give readers additional information about their work.

© 2023 Hazra A et al. *JAMA Network Open.*

eMethods

Data Source. In 1996 the Ministry of Health launched the Jordan Cancer Registry (JCR). JCR is a national, population-based registry. Cancer data is collected from all hospitals, clinical pathology labs, and hematology labs in Jordan. The JCR uses active and passive methods of case finding from hospitals and clinical labs. In the Active method of data collection and case finding, the registry staff visits the medical facilities. The Passive method sends a complete notification form to the JCR. The data is coded using ICD-O3 CanReg_4 and CanReg_5 software for data processing and analysis. Refugees presenting at hospitals or clinics are required to present personal identification, such as UN High Commission for Refugees (UNHCR) identification cards and Ministry of the Interior (MOI) Interior Service Card.

This study followed the Strengthening the Reporting of Observational Studies in Epidemiology (STROBE)-Equity reporting guidelines.

We retrospectively queried the JCR for deidentified data on breast cancer diagnosed from January 1, 2010, to December 31, 2016. In this cross-sectional, secondary analysis of cancer registry data, Syrian migrant women were defined as Syrian nationals based on the UNHCR and MOI cards presented at the healthcare facility. These data were coded in the JCR. These Syrian nationals resided in the refugee camps or in urban settings in Jordan. We excluded five Syrian migrant men and 114 Jordanian men from the current analysis. Our de-identified dataset includes 7,891 women diagnosed with breast cancer, coded as International Classification of Diseases, Tenth Revision (ICD-10) C50.

© 2023 Hazra A et al. *JAMA Network Open.*

Variables. Epidemiological data (gender, age, smoking status, marital status), tumor characteristics (date of diagnosis, primary site, histology, behavior, grade, and SEER summary stage), and clinical covariates (date of last contact, death) were recorded in the JCR. The abstracted data were coded according to the international classification of disease ICD-10 morbidity and mortality coding system. The primary site (topography) and histology (morphology) of the malignancies are identified and coded according to the International Classification of Diseases for Oncology (ICD-O), published by the WHO in 2018. Cases with a tumor behavior code of 2: in situ or 3: invasive in the ICD-O-3 are included in the registry and in the analysis.

Clinical stages of breast cancer at presentation were classified according to the National Cancer Institute's (NCI) Surveillance, Epidemiology, and End Results (SEER) Summary Stage codes 0: intraepithelial, non-invasive, or non-infiltrating cancer is described as "*in situ*"; 1: "localized" tumor confined to the breast without direct extension beyond the breast; 2: "regional extension" of tumor can by direct extension to adjacent organs only; 3: "regional" extension by lymph node involvement only; 4: "regional extension" of the tumor by both direct extension and lymph node involvement; 5: "regional, NOS"; 7: "distant metastasis", and 9: "unknown" stage is coded when there insufficient information to assign a stage (and missing when no code is recorded).

Statistical Analysis. Registry data for Syrian migrants and Jordanians were merged and analyzed using R software (https://www.r-project.org). Covariates, including age at diagnosis, diagnosis date, sex, nationality, tumor behavior, morphology, and topology had complete case data. Missing data coded as "9 or 99" in the registry for other categorical

© 2023 Hazra A et al. *JAMA Network Open.*

variables were recoded as “NA”. A category for “unknown” data is included in Table 1. The six common morphology categories are shown in Table 1, and the less common categories were grouped as “other”. Associations of migrant status (coded as a binary variable for Syrian migrants and refugees or Jordanian) or above or below median age (coded as a binary variable ≤49, ≥50) with categorical lifestyle factors and tumor features were evaluated using Chi-square (χ^2), Fisher exact, and Kruskal-Wallis tests to assess significance (in cases with known data). We used the logit model to model the log odds of being diagnosed with late-stage breast cancer (coded as a binary variable for distant metastasis versus localized-regional disease). All statistical tests were two-sided. P-values <0.05 were considered statistically significant.

© 2023 Hazra A et al. *JAMA Network Open.*
