# Supplementary material for: Disparities in Breast Cancer Characteristics Among Syrian Migrants and Jordanian Women in the Jordan Cancer Registry from 2010 to 2016
Source: JAMA Netw Open. 2023 Jul 24;6(7):e2325197. doi: 10.1001/jamanetworkopen.2023.25197 (PMC10366694; doi:10.1001/jamanetworkopen.2023.25197)
Supplement: Supplement 2. — Data Sharing Statement [file jamanetwopen-e2325197-s002.pdf]

Data Sharing Statement

Hazra. Disparities in Breast Cancer Characteristics Among Syrian Migrants and Jordanian Women in the Jordan Cancer Registry from 2010 to 2016. *JAMA Netw Open*. Published July 24, 2023. doi:10.1001/jamanetworkopen.2023.25197

Data
Data available: No

Additional Information
Explanation for why data not available: The registry data is available through the Ministry of Health. The retrospective study only included de-identified cancer registry data. This study was exempt from informed consent because identifiable information was not included in the dataset. Privacy of refugee women is protected.
